# Accuracy of Patient Opioid Use Reporting at the Time of Medical Cannabis License Renewal

**DOI:** 10.1155/2018/5704128

**Published:** 2018-01-28

**Authors:** Jacob M. Vigil, Sarah S. Stith, Anthony P. Reeve

**Affiliations:** ^1^Department of Psychology, University of New Mexico, Albuquerque, NM, USA; ^2^Department of Economics, University of New Mexico, Albuquerque, NM, USA; ^3^Industrial Rehabilitation Clinics, Albuquerque, NM, USA

## Abstract

The decision to authorize a patient for continued enrollment in a state-sanctioned medical cannabis program is difficult in part due to the uncertainty in the accuracy of patient symptom reporting and health functioning including any possible effects on other medication use. We conducted a pragmatic convenience study comparing patient reporting of previous and current prescription opioid usage to the opioid prescription records in the Prescription Monitoring Program (PMP) among 131 chronic pain patients (mean age = 54; 54% male) seeking the first annual renewal of their New Mexico Medical Cannabis Program (NMMCP) license. Seventy-six percent of the patients reported using prescription opioids prior to enrollment in the NMMCP, however, the PMP records showed that only 49% of the patients were actually prescribed opioids in the six months prior to enrollment. Of the 64 patients with verifiable opioid prescriptions prior to NMMCP enrollment, 35 (55%) patients reported having eliminated the use of prescription opioids by the time of license renewal. PMP records showed that 26 patients (63% of patients claiming to have eliminated the use of opioid prescriptions and 41% of all patients with verifiable preenrollment opioid use) showed no prescription opioid activity at their first annual NMMCP renewal visit.

## 1. Introduction

The current opioid epidemic has resulted in the immediate need for alternative pain treatments [1, 2], such as medical cannabis [3–5], which has been authorized in one form or another in most of the sates of the U.S. However, the decision to authorize a patient for enrollment in a state-sanctioned medical cannabis program can be challenging due to the dearth of extant clinical research on the likely health outcomes of enrollment for patients overall, as well as for potentially higher risk subgroups such as older patients. Another uncertainty is the accuracy of symptom reporting by patients often on prescription opioids and seeking to obtain legal access to an additional addictive, controlled substance that is widely used for nonmedical purposes in the general population and hence has the potential for nonmedical abuse and diversion.

In our (APR) rehabilitation clinic, where we authorize patients for initial and continued enrollment in the New Mexico Medical Cannabis Program (NMMCP), we noticed a pattern recently described in the literature [6, 7], whereby a number of patients claimed to have replaced their prescription opioids in favor of using cannabis for treating chronic pain. In order to assess whether those anecdotal reports were, in fact, true and occurring over a broader spectrum of patients rather than simply in a few incidental cases, we conducted a pragmatic convenience study among people authorized to use cannabis for treating a chronic pain condition comparing patient reporting to their Prescription Monitoring Program (PMP) opioid prescription records.

## 2. Methods

One year following enrollment in the NMMCP, 131 patients (mean age = 54; 54% male, 65% chronic back pain, 27% other chronic musculoskeletal pain, 4% fibromyalgia; 2% arthritis, and 2% chronic headaches) from a single rehabilitation clinic in Albuquerque, NM, completed a brief survey asking (1) whether or not they were using prescription opioids prior to enrollment in the NMMCP (“When you began the program, did you use narcotics for pain management?”) and (2) if they were able to cease the use of all prescription opioids by the time of renewal (after 12 months enrolled in the NMMCP). While enrolled in the NMMCP, patients received no direct medical supervision over their cannabis usage (e.g., frequency, dosage, or type of cannabis product), nor were they provided any explicit instructions for modifying (e.g., reducing) their opioid usage. Preenrollment prescription opioid use was verified with PMP evidence of any opioid prescriptions in the 6 months leading up to enrollment in order to capture recent prescription activity, and current opioid use was verified with evidence of any opioid prescription in month 12 or 13 after enrollment.

## 3. Results

**Figure 1:**
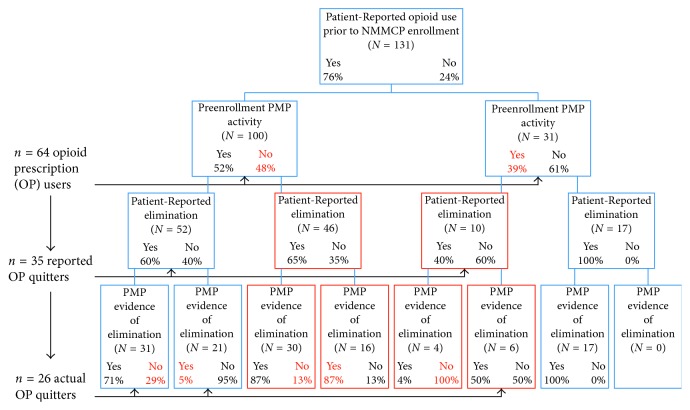
Diagram of patient-reported and PMP-recorded opioid prescriptions. The percentages refer to the proportion of that subsample (*N* ) responding as specified. “Patient-reported” refers to patients' queried responses at the time of NMMCP enrollment renewal; “PMP” refers to categorizations based on evidence in the PMP records. Patients were asked about preenrollment use. PMP records covering the six month period prior to enrollment were used to measure preenrollment PMP activity; PMP records for months 12 and 13 were used to evaluate elimination. Red-colored patient rates indicate misreporting; red outlines indicate repeated misreporting.

Figure 1 shows that over three-fourths (76%) of the patients we observed reported using prescription opioids prior to enrollment in the NMMCP; however, the PMP records showed that only half (49%) of the patients were actually prescribed opioids in the six months prior to enrollment. Of the 64 patients with verifiable opioid prescriptions prior to MCP enrollment, 35 (55%) patients reported having eliminated the use of prescription opioids (in favor of using cannabis for treating their chronic pain conditions) by the time of MCP enrollment renewal. PMP records show that 22 (63%) of these patients did, indeed, show no PMP evidence of opioid prescriptions in months 12-13 after enrollment, and an additional 4 patients that misreported *not* eliminating prescription opioid usage actually showed no verifiable records of opioid prescriptions. This resulted in a final count of 26 patients (41% of all patients with verifiable preenrollment opioid use) showing no PMP records of opioid prescriptions at the time of their first annual MCP renewal visit. Follow-up independent samples *t*-tests revealed, unlike other studies showing a greater preponderance of misreporting medication use among males [8], no gender or age differences (contrasting patients above and below 55 yrs) in the accuracy of patient-reported opioid prescription use among patients claiming to have eliminated the use of prescription opioids (*p'*s = 0.629 and 0.901, resp.).

## 4. Discussion

Misuse, underreporting, and noncompliance of medication use among chronic pain patients are notoriously common [9–14]; however, at least in the context of seeking a renewal of their medical cannabis license, the rates of misreporting the ability to substitute the use of opioids in favor of medical cannabis appeared to be relatively low. In addition to documenting the consistent accuracy of patient opioid use reporting in the context of medical cannabis license renewal, the patterns in the PMP data support exploring the possibility of using medical cannabis to treat chronic pain as a substitute for opioids. Colocalization and potential synergistic effects of *µ*-opioid and CB1 receptors in various regions of the central nervous system make the adjunct use of raw natural cannabis and cannabis formulations viable options for future research on their analgesic therapeutic potential [3–5, 15]. Recent studies have shown that state-enacted laws enabling patients to legally obtain and use medical cannabis are associated with significant (up to 33%) reductions in opioid-related deaths in those regions [16, 17]. Contributing to a growing literature that shows an association between significant reductions in opioid prescriptions and medical cannabis [6, 18], we found that the majority of patients reporting to be using medical cannabis instead of prescription opioids for treating their chronic pain have PMP records consistent with this claim. Our pragmatic observation study using a convenience sample is superior to nonrepresentative samples used in RCTs, because they do not capture actual use of opioids and cannabis among “real” patients. Of course, the obvious limitations of our approach are that opioid prescribing does not automatically equate to patient opioid use (e.g., diversion) and conversely a previous prescription could be used by patients at a later date to obtain opioids. No study to date has compared the long-term effects of using prescription opioids *versus* medical cannabis for treating chronic pain. However, from a harm reduction standpoint considering the emergent opiate crisis in this U.S. along with the notable risks of abuse [14] and overdose [19, 20], alternative treatments for chronic pain need to be explored and researched, and medical cannabis may be a viable alternative. Unfortunately, federal barriers continue to exist which largely restrict the ability to conduct practical medical cannabis research [21].

## Abbreviations

PMP: Prescription Monitoring Program
NMMCP: New Mexico Medical Cannabis Program.
